# Cryoglobulinemia in systemic lupus erythematosus: a retrospective study of 213 patients

**DOI:** 10.1186/s13075-022-02857-z

**Published:** 2022-07-14

**Authors:** Yoann Roubertou, Sabine Mainbourg, Arnaud Hot, Denis Fouque, Cyrille Confavreux, Roland Chapurlat, Sébastien Debarbieux, Denis Jullien, Pascal Sève, Laurent Juillard, Marie-Nathalie Kolopp-Sarda, Jean-Christophe Lega

**Affiliations:** 1 Hospices Civils de Lyon, Department of Internal and Vascular Medicine, Centre Hospitalier Lyon Sud, Lyon, France; 2 Université Lyon I, Lyon, France; 3 Hospices Civils de Lyon, Lyon Immunopathology Federation, Université Lyon I, Lyon, France; 4 UMR 5558, Equipe Evaluation et Modélisation des Effets Thérapeutiques, Laboratoire de Biométrie et Biologie Evolutive, CNRS, Université Lyon I, Lyon, France; 5 Department of Internal Medicine, Hospices Civils de Lyon, Hôpital Edouard Herriot, Lyon, France; 6 Department of Nephrology, INSERM U1060 CarMeN, Hospices Civils de Lyon, Centre Hospitalier Lyon-Sud, Université Lyon I, Lyon, France; 7 Department of Rheumatology, Hospices Civils de Lyon, Centre Hospitalier Lyon Sud, Lyon, France; 8 Department of Rheumatology, Hospices Civils de Lyon, Hôpital Edouard Herriot, Lyon, France; 9 Department of Dermatology, Centre Hospitalier Lyon Sud, Hospices Civils de Lyon, Lyon, France; 10 Department of Dermatology, Hospices Civils de Lyon, Hôpital Edouard Herriot, Lyon, France; 11 Department of Internal Medicine, Hospices Civils de Lyon, Hôpital de la Croix-Rousse, Lyon, France; 12 Department of Nephrology, Hospices Civils de Lyon, Hôpital Edouard Herriot, Lyon, France; 13 Immunology Laboratory, Hospices Civils de Lyon, Centre Hospitalier Lyon Sud, Lyon, France

**Keywords:** Systemic lupus erythematosus, Cryoglobulinemia, Cryoglobulinemic vasculitis

## Abstract

**Objectives:**

The clinical value of cryoglobulinemia (CG) in systemic lupus erythematosus (SLE) is largely unknown. The aim of this retrospective study was to describe the characteristics of CG in SLE, its impact on SLE phenotype, and the features associated with cryoglobulinemic vasculitis (CryoVas) in SLE patients.

**Methods:**

This retrospective study conducted in a French university hospital reviewed the data from 213 SLE patients having been screened for CG between January 2013 and December 2017. SLE patients positive for CG were compared to SLE patients without CG. Patients were classified as CryoVas using the criteria of De Vita et al.

**Results:**

Of the 213 SLE patients included (mean age 29.2 years, female sex 85%), 142 (66%) had at least one positive CG in their history, 67% of them having a persistent CG at follow-up. CG was type III in 114 (80%) cases and type II in 27 (19%) cases. The mean concentration of the cryoprecipitate was 40mg/L (range 0-228). Patients with CG had significantly more C4 consumption. Among patients with CG, 21 (15%) developed a CryoVas. The clinical manifestations of patients with CryoVas were mainly cutaneous (purpura, ulcers, digital ischemia) and articular, without any death at follow-up. Severe manifestations of CG included glomerulonephritis in 1/21 (5%) patients and central nervous system involvement in 4/21 (19%) patients. A response to first-line treatments was observed in 12/13 (92%) patients, but relapses were observed for 3 of them.

**Conclusion:**

CG is frequent in SLE, but mostly asymptomatic. CryoVas features involve mostly joints, skin, and general symptoms. CryoVas in SLE appears to be a specific condition, with a low prevalence of neuropathy, membranoproliferative glomerulonephritis, and severe manifestations.

**Supplementary Information:**

The online version contains supplementary material available at 10.1186/s13075-022-02857-z.

## Highlights

• Cryoglobulinemia is frequent in SLE, but mostly asymptomatic.

• Sixty-six percent of SLE patients tested positive for cryoglobulins, and 15% of the SLE patients with cryoglobulinemia developed a cryoglobulinemic vasculitis.

• Features of the cryoglobulinemic vasculitis mainly involved skin, joints, and general signs. Severe manifestations of vasculitis were rare.

**Supplementary Information:**

The online version contains supplementary material available at 10.1186/s13075-022-02857-z.

## Introduction

Systemic lupus erythematosus (SLE) is one of the most frequent connective tissue diseases and affects predominantly young women [1, 2]. Its clinical presentation is heterogeneous and can involve many organs such as skin, joints, kidney, central nervous system, and blood cells. The SLE biological autoimmune disorders include the presence of antinuclear antibodies (ANA) in almost all patients and double-stranded DNA (dsDNA) antibodies in two-thirds of patients, usually associated with low complement levels [3]. Other immunological features include cryoglobulin in 19-83% of patients [4–12].

Cryoglobulins are immunoglobulins characterized by reversible precipitation at a low temperature. The main clinical consequences of cryoglobulinemia (CG) are due to small-vessel vasculitis inducing cutaneous, renal, articular, and neurological involvements [13]. CG is associated with B cell lymphoproliferative diseases, chronic infections (mainly hepatitis C virus, HCV), and autoimmune diseases. In 1974, Brouet et al. proposed a classification of CG according to its monoclonal (type I) and/or polyclonal (types II and III) composition [14]. SLE is one of the most frequent autoimmune diseases associated with CG along with Sjogren’s syndrome [15].

The data in the literature concerning the phenotype of SLE patients with CG is scarce and not up-to-date, and the clinical impact of CG on SLE remains poorly described [10, 16]. The few studies published are limited in terms of external validity (out-of-date), inclusion of HCV patients [16], and sample size [10].

This retrospective cohort study aimed to describe the clinical and biological phenotype of SLE patients positive for CG, including patients with a cryoglobulinemic vasculitis (CryoVas).

## Material and methods

### Patient selection and study design

A retrospective study was conducted in a tertiary care center. The data of all patients who had ANA ≥1/160 and at least one positive antibody among dsDNA antibodies, anti-Sm antibodies, or anti-SSA antibodies, between January 1, 2013, and December 31, 2017, were retrospectively analyzed. Patients without CG research or patients positive for HCV were excluded. Medical charts were reviewed to assess the diagnosis of SLE according to the Systemic Lupus International Collaborating Clinics (SLICC) criteria [17] and/or the European League Against Rheumatism (EULAR) 2019 criteria [18].

The SLE patients were classified according to their CG status. The first group included SLE patients with the presence of CG reported at least once in their history (SLE CG+). The second group included SLE patients with negative CG reported in their history (SLE CG−). The clinical and biological data of the included patients were considered from the time of SLE diagnosis until June 30, 2020.

The database was reported to the national data protection agency (*Commission Nationale de l’Informatique et des Libertés*, CNIL) and the study was approved by the ethics committee of the *Hospices Civils de Lyon* (validation number 21_5338). Patients were informed and could express their refusal to participate in the study.

### Clinical variables

Data for demographic, clinical, and laboratory items were collected using standardized forms. The date of SLE diagnosis was registered for each patient. Clinical manifestations of SLE during the medical history were collected. Specific treatments including steroids, immunosuppressive, immunomodulatory, and biologic therapies were investigated as well as their medical indication (SLE or CryoVas). SLE disease activity was assessed retrospectively using the SLE Disease Activity Index (SLEDAI) [19] at SLE diagnosis, at the date of first positive CG, and at the date of an eventual CryoVas. A flare was defined by an SLEDAI >4. Clinical data potentially linked to CG at the time of the first positive CG were collected. The dates of the first positive CG and CryoVas were registered. Patients were classified as having CryoVas using the 2011 criteria of De Vita et al. [20], involving clinical and laboratory items. Clinical manifestations of the CryoVas were collected. Patients were classified as having central nervous system involvement in case of cerebral vasculitis, myelitis, or meningoencephalitis attributed to the CryoVas. If a biopsy of skin, kidney, or peripheral nervous system was performed, a histology compatible with CryoVas was searched for. Deaths of any cause and loss to follow-up were registered. Cardiovascular events (acute coronary syndrome, stroke) and severe infections (intravenous antimicrobial treatment or hospitalization for infection) were collected as well as their subtypes.

### Laboratory parameters

Cryoglobulin tests, purification, and characterization were performed in the immunology laboratory of the *Hospices Civils de Lyon* according to the local protocol previously published [21]. Blood samples were collected by venipuncture for cryoglobulin testing and complement exploration. Samples were then rapidly sent to the laboratory at 37°C and maintained in an incubator at 37°C to clot for a minimum of 2 hours. Samples were centrifuged and serum was preserved at 4°C for 7 days. Visual observation at day 7 allowed the detection of any cryoprecipitate. In that case, cryoprecipitates were isolated by +4°C centrifugation (2200*g*, 15 min) and purified by 3 washes with cold phosphate-buffered saline (PBS, pH 7.4, +4°C) to remove serum and proteins which had not precipitated. Pellets were then dissolved at 37°C in 500 μL PBS and conserved at 37°C for further analysis. Characterization of the cryoprecipitate was performed by electrophoresis-immunofixation to type cryoglobulins with anti-γ, anti-α, anti-μ, anti-λ and anti-κ antisera (SAS-3®, Helena Bioscience, Gateshead, UK). In the dissolved cryoprecipitate conserved at 37°C, IgG, IgM, and/or IgA concentrations as well as rheumatoid factor (RF) activity (anti-IgG IgM) were assayed by immunonephelemetry (BNprospec®, Siemens, Marburg, Germany). Serum RF (normal <20UI/mL), complement C3 (normal range 0.8–1.58 g/L) and C4 (normal range 0.16–0.39 g/L) were quantified by immunonephelometry (BNprospec®), and complement functional activity (CH50) was quantified on Optilite® (The Binding Site, Birmingham, UK; normal range 41–95 U/mL).

Anti-dsDNA antibodies were determined using a radioimmunological test (Farr test anti-dsDNA, Trinity Biotech, Wicklow, Ireland) with a cut-off at 10 IU/mL. ANA (indirect immunofluorescence on HEp2 cells, Biorad®, Hercules, CA, USA), complement, anti-dsDNA test, gammaglobulins, and RF levels were assessed at the time of the first positive CG or 3 months before or after the first research for CG if no CG was documented. The other autoimmune parameters (anti-RNP, SSA, Sm, CCP, antiphospholipid antibodies) were registered as positive if persistent across the medical history of the patient and if the levels were superior to the laboratory’s reference ranges. Antiphospholipid antibodies were registered as positive if persistent at least 12 weeks later, following Sapporo criteria for antiphospholipid syndrome [22].

The viral status against hepatitis B virus (HBV), HCV, and human immunodeficiency virus (HIV) was also registered. HBV status was considered positive if HbS antigen was present, and HCV status was considered positive if anti-HCV antibodies were present, regardless of the viral load status. The other laboratory parameters (urine sediment, creatinine levels, blood cell counts, etc.) were determined using the routine procedures of the *Hospices Civils de Lyon* laboratory. Lymphopenia was considered if total lymphocytes were inferior to 1 G/L, leukopenia if white cell counts were inferior to 4 G/L, and thrombocytopenia if platelets were inferior to 100.10^9^/L. These parameters had to be present at least twice and persistent at a minimum of 12 weeks during the medical history to be registered.

### Statistical analysis

Baseline characteristics were assessed using descriptive statistics. Continuous variables were described by their means and standard deviations and compared using *t*-tests. Categorical variables were described as numbers and percentages and compared with *χ*^2^ or Fisher’s exact test. For all statistical analyses, the Bonferroni correction was applied to control for type I errors and statistical significance was set at *p*<0.0003. Analyses were performed using R (R Foundation for Statistical Computing, Vienna, Austria)

## Results

### Studied population

Among ANA-positive patients, a sample of 224 patients with SLE was identified. Three patients were excluded because of HCV infection and 8 were excluded due to the absence of CG testing in the medical file. After reviewing medical charts, 213 patients with SLE were included in the study, 85% of them being women. The mean age at SLE diagnosis was 29.2 (±12.8) years. The mean duration of follow-up was 13.2 (±8.9) years. CG was positive at least once in 142/213 patients (66%; Table 1). Twenty-one (10%) patients were lost to follow-up before June 30, 2020 (12 from the SLE CG+ group and 9 from the SLE CG− group). Two patients died (one in each group).

**Table 1:** SLE clinical manifestations according to the presence of cryoglobulinemia

	SLE CG+ (***n*** = 142)	SLE CG− (***n*** = 71)	***p*** value
Female sex, *n* (%)	124 (87%)	56 (79%)	0.11
**SLE diagnosis**			
Age at SLE diagnosis, (years), mean (± SD)	29.1 ± 12.3	28.1 ± 13.7	0.15
SLEDAI score at SLE diagnosis, mean (± SD)	14.2 ± 7.5	12.5 ± 7.5	0.051
**SLE clinical manifestations**			
Acute cutaneous lupus, *n* (%)	69 (49%)	34 (48%)	0.92
Subacute cutaneous lupus, *n* (%)	41 (29%)	13 (18%)	0.10
Chronic cutaneous lupus, *n* (%)	26 (18%)	11 (15%)	0.61
Oral ulcers, *n* (%)	29 (20%)	13 (18%)	0.72
Alopecia, *n* (%)	36 (25%)	15 (21%)	0.50
Cutaneous vasculitis unrelated to CG, *n* (%)	1 (1%)	8 (11%)	0.0008
Raynaud’s phenomenon, *n* (%)	70 (49%)	27 (38%)	0.12
Arthritis and/or arthralgia, *n* (%)	132 (93%)	65 (92%)	0.71
Pericarditis, *n* (%)	34 (24%)	16 (23%)	0.82
Myocarditis, *n* (%)	7 (5%)	3 (4%)	1.00
Pleuritis, *n* (%)	19 (13%)	11 (15%)	0.68
Cardiac valvulopathy, *n* (%)	9 (6%)	2 (3%)	0.85
Intra-alveolar hemorrhage, *n* (%)	2 (1%)	3 (4%)	0.62
Pulmonary hypertension, *n* (%)	2 (1%)	3 (4%)	0.84
Nephritis, *n* (%)	64 (45%)	27 (38%)	0.33
Peripheral nervous system, *n* (%)	4 (3%)	3 (4%)	1.00
Central nervous system, *n* (%)	17 (12%)	11 (15%)	0.47
Psychosis, *n* (%)	27 (19%)	13 (18%)	0.91
**Associated autoimmune disease**			
Antiphospholipid syndrome, *n* (%)	37 (26%)	11 (15%)	0.08
Sjogren’s syndrome, *n* (%)	28 (20%)	11 (15%)	0.45
Other connective tissue disease, *n* (%)	14 (10%)	2 (3%)	0.07

*SLE* Systemic lupus erythematosus, *SLEDAI* Systemic lupus erythematosus activity index, *CG* Cryoglobulinemia

### Immunological characteristics of the cryoglobulinemia

The CG was mostly type III (114/142, 80%), and 73/142 (51%) patients with type III CG had polyclonal IgG and polyclonal IgM. The mean total Ig concentration of the CG was 40mg/L (range 0–228; Table 2). RF activity in the cryoprecipitate was negative for 125/142 (88%) patients. Six (4%) of the 142 patients had a positive RF in the serum and 3 (2%) had a positive RF in the cryoprecipitate (Table 2). For 14 patients, the CG isotype was not available because of old data. For the specific CryoVas group, the mean total concentration of the cryoprecipitate was 31.1mg/L (range 8.6–81.8). The proportion of CG type II and CG type III was not significantly different between the CryoVas group and the SLE CG+ without the CryoVas group.

**Table 2:** Immunological characteristics of the cryoglobulin in SLE patients

	SLE CG+ (***n*** = 142)
Type I cryoglobulinemia, *n* (%)	1 (1%)
Type II cryoglobulinemia, *n* (%)	27 (19%)
Type III cryoglobulinemia, *n* (%)	114 (80%)
**Ig concentration in cryoprecipitate (mg/L)**	
Total, mean (range)	40 (0-228)
IgG, mean (range)	21 (0-107)
IgA, mean (range)	1 (0-27)
IgM, mean (range)	19 (0-228)
**Type I cryoglobulinemia**	
Monoclonal IgMκ, *n* (%)	1 (1%)
**Type II cryoglobulinemia isotypes**	
Monoclonal IgMκ + polyclonal IgG/IgM, *n* (%)	15 (11%)
Monoclonal IgGκ + polyclonal IgG/IgM, *n* (%)	4 (3%)
Monoclonal IgMλ + polyclonal IgG/IgM, *n* (%)	4 (3%)
Monoclonal IgGλ + polyclonal IgG/IgM, *n* (%)	2 (1%)
**Type III cryoglobulinemia isotypes**	
Polyclonal IgG + polyclonal IgM, *n* (%)	73 (51%)
Polyclonal IgG, *n* (%)	22 (15%)
Polyclonal IgG + polyclonal IgM + polyclonal IgA, *n* (%)	3 (2%)
Polyclonal IgM, *n* (%)	1 (1%)
**RF activity**	
Negative RF in cryoprecipitate, *n* (%)	125 (88%)
Positive RF in cryoprecipitate, *n* (%)	3 (2%)

*Ig* Immunoglobulins, *RF* Rheumatoid factor

Within the SLE CG+ group, 96/142 (67%) patients had a persistent CG, 32/142 (23%) patients had a transient CG, and 14/142 (10%) had a positive CG without additional confirmatory testing.

### SLE manifestations according to the presence of cryoglobulinemia

There was no significant difference between the SLE CG+ and SLE CG− groups concerning nephritis, arthritis, dermatitis, and Raynaud’s phenomenon.

The frequency of associated autoimmune diseases was not significantly different between SLE CG+ and SLE CG− groups.

In the SLE-CG+ compared to the SLE CG− group, there was a significantly higher frequency of decreased C4 (75% versus 46%, respectively, *p*=0.00003) (Table 3).

**Table 3:** SLE laboratory features in SLE patients according to the presence of a cryoglobulin

	SLE CG+ (***n*** = 142)	SLE CG− (***n*** = 71)	***p*** value
**Immunology**			
RF in serum positive, *n* (%)	6 (4%)	5 (7%)	0.51
RF in serum, UI/L, mean (± SD)	119 ± 100	107 ± 110	0.69
Hypogammaglobulinemia, *n* (%)	6 (4%)	3 (4%)	1.00
Hypergammaglobulinemia, *n* (%)	87 (61%)	34 (48%)	0.06
Gammaglobulins, g/L, mean (± SD)	16.2 ± 6.0	16.8 ± 7.0	0.89
Decreased complement C3, *n* (%)	96 (68%)	32 (45%)	0.002
Decreased complement C4, *n* (%)	107 (75%)	33 (46%)	<0.00013
Decreased CH50, *n* (%)	91 (64%)	28 (39%)	0.0006
Farr test, UI/L, mean (± SD)	72.2 ± 88.0	42.7 ± 51.2	0.07
Anti-SSA antibodies, *n* (%)	72 (51%)	32 (45%)	0.44
Anti-Sm antibodies, *n* (%)	55 (39%)	22 (31%)	0.27
Anti-RNP antibodies, *n* (%)	65 (46%)	27 (38%)	0.28
**Infections**			
HBV infection, *n* (%)	2 (1%)	0 (0%)	0.90
HIV infection, *n* (%)	0 (0%)	0 (0%)	1.00
**Cytopenia**			
Leukopenia, *n* (%)	36 (25%)	22 (31%)	0.38
Lymphopenia, *n* (%)	89 (63%)	40 (56%)	0.37
Thrombopenia, *n* (%)	36 (25%)	18 (25%)	1.00
Autoimmune hemolytic anemia, *n* (%)	23 (16%)	9 (13%)	0.50

*RF* Rheumatoid factor, *HBV* Hepatitis B virus, *HIV* Human immunodeficiency virus

### Clinical signs related to cryoglobulinemia

In the SLE CG+ group, the first positive CG was found at a mean of 6.4 years after the diagnosis of SLE. The specific signs of vasculitis in the SLE CG+ group were mainly cutaneous manifestations, including purpura, ulcers, digital ischemia, and livedo (Table 4).

**Table 4:** Clinical signs associated with cryoglobulinemia

	SLE CG+ (***n*** = 142)
**Cryoglobulinemia diagnosis**	
Years between SLE diagnosis and first CG, mean (±SD)	6.4 ± 7.4
Years between SLE diagnosis and first clinical signs linked to CG, mean (±SD)	6.6 ± 9.7
SLEDAI score at SLE diagnosis, mean (±SD)	14.2 ± 7.5
SLEDAI score at CG diagnosis, mean (±SD)	14.9 ± 8.9
**Vasculitis features at the time of the first positive CG**	
Purpura, *n* (%)	9 (6%)
Acrocyanosis, *n* (%)	15 (11%)
Digital ischemia, *n* (%)	9 (6%)
Cutaneous necrosis, *n* (%)	9 (6%)
Ulcers, *n* (%)	16 (11%)
Livedo, *n* (%)	23 (16%)

*CG* Cryoglobulinemia, *SLEDAI* Systemic lupus erythematosus activity index

### Cardiovascular events and severe infections in SLE patients according to the presence of cryoglobulinemia

No significant difference was observed between SLE CG+ and SLE CG− groups regarding cardiovascular events (14% versus 10%, respectively) or severe infections (26% versus 23%, respectively, see supplemental Table 1).

### Characteristics of patients with CryoVas

In the SLE CG+ group, 21/142 (15%) patients presented a CryoVas according to de Vita’s criteria [20], and 7/21 were documented with biopsy findings of histological vasculitis (skin biopsy=5, nerve biopsy=1, muscle biopsy=1). No kidney biopsy retrieved lesions of membranoproliferative glomerulonephritis, and no death occurred. The delay between SLE diagnosis and the first positive test for CG was significantly longer in the CryoVas group than in the group without CryoVas (7.6 years versus 5.9 years, respectively, *p*<0.0001).

Concerning clinical signs attributed to CG, patients with CryoVas had significantly more purpura (43% versus 0%, *p* <0.00001) and cutaneous necrosis (38% versus 1%, *p*<0.0001). In the CryoVas group, severe manifestations included kidney failure and central nervous system involvement in 1/21 (5%) and 4/21 (19%) patients, respectively. Central nervous system involvement in CryoVas patients included strokes and cerebral vasculitis. Moreover, patients with CryoVas presented significantly more pericarditis across their SLE medical history (52% versus 19%, *p*<0.0001). The proportion of lupus nephritis was not significantly different between patients with CryoVas and those without (43% vs 45%, respectively, *p*=0.97; Table 5).

**Table 5:** Characteristics of SLE patients with cryoglobulinemia according to CryoVas status

	SLE CG+ with CryoVas (***n*** = 21)	SLE CG+ without CryoVas (***n*** = 121)	***p*** value
Female sex, *n* (%)	16 (76%)	108 (89%)	0.15
**SLE and CG diagnosis**			
Age at lupus diagnosis, (years), mean (±SD)	28.0 ± 14.1	30.0 ± 12.0	0.54
Years between SLE diagnosis and first positive CG, mean (±SD)	7.6 ± 9.1	5.9 ± 7.1	<0.0001
Years between SLE and CryoVas diagnosis, mean (±SD)	8.5 ± 9.7	NA	
SLEDAI score at SLE diagnosis, mean (±SD)	17.0 ± 7.5	13.6 ± 7.4	0.03
SLEDAI score at CG diagnosis, mean (±SD)	17.9 ± 9.0	14.3 ± 8.9	0.08
SLEDAI score at CryoVas diagnosis, mean (±SD)	23.7 ± 9.9	NA	
**Associated autoimmune disorder**			
Antiphospholipid syndrome, *n* (%)	7 (33%)	30 (25%)	0.42
Sjogren’s syndrome, *n* (%)	4 (19%)	24 (20%)	1.00
**SLE clinical manifestations**			
Acute cutaneous lupus, *n* (%)	13 (62%)	56 (46%)	0.19
Subacute cutaneous lupus, *n* (%)	10 (48%)	31 (26%)	0.04
Chronic cutaneous lupus, *n* (%)	5 (24%)	21 (17%)	0.54
Oral ulcerations, *n* (%)	5 (24%)	24 (20%)	0.54
Alopecia, *n* (%)	9 (43%)	27 (22%)	0.046
Raynaud’s phenomenon, *n* (%)	15 (71%)	55 (45%)	0.03
Arthritis and/or arthralgia, *n* (%)	21 (100%)	111 (92%)	0.36
Pericarditis, *n* (%)	11 (52%)	23 (19%)	<0.0001
Myocarditis, *n* (%)	2 (10%)	5 (4%)	0.28
Pleuritis, *n* (%)	2 (10%)	17 (14%)	0.74
Valvulopathy, *n* (%)	3 (14%)	6 (5%)	0.13
Intra-alveolar hemorrhage, *n* (%)	1 (5%)	1 (1%)	0.27
Pulmonary arterial hypertension, *n* (%)	0 (0%)	1 (1%)	1.00
Nephritis, *n* (%)	9 (43%)	55 (45%)	0.97
Peripheral nervous system, *n* (%)	2 (10%)	2 (2%)	0.08
Central nervous system, *n* (%)	3 (14%)	14 (12%)	0.72
Psychosis, *n* (%)	5 (24%)	22 (18%)	0.55
Leukopenia, *n* (%)	6 (29%)	30 (25%)	0.71
Lymphopenia, *n* (%)	15 (71%)	74 (61%)	0.37
Thrombopenia, *n* (%)	6 (29%)	30 (25%)	0.71
Autoimmune hemolytic anemia, *n* (%)	3 (14%)	20 (17%)	1.00
**Immunology**			
RF in serum positive, *n* (%)	1 (5%)	5 (4%)	1.00
RF in serum, UI/L, mean (±SD)	25 ± 4.2	137 ± 100	0.02
RF in cryoglobulin positive, *n* (%)	0 (0%)	3 (2%)	1.00
Hypogammaglobulinemia, *n* (%)	0 (0%)	6 (5%)	0.59
Hypergammaglobulinemia, *n* (%)	16 (76%)	71 (59%)	0.13
Gammaglobulins, g/L, mean (±SD)	17.4 ± 4.0	15.9 ± 5.9	0.17
Decreased complement C3, *n* (%)	17 (81%)	79 (65%)	0.16
Decreased complement C4, *n* (%)	19 (90%)	88 (73%)	0.08
Decreased CH50, *n* (%)	17 (81%)	74 (61%)	0.08
Farr test, UI/L, mean (±SD)	86.4 ± 82.1	69.9 ± 86.3	0.48
**Total concentration of cryoprecipitate, mg/L, mean (range)**	31.1 (8.6-81.8)	41.2 (6.1-228)	0.06
**Type I cryoglobulinemia**, *n* (%)	0 (0%)	1 (1%)	
**Type II cryoglobulinemia**, *n* (%)	4 (19%)	23 (19%)	1.00
Monoclonal IgMκ + polyclonal IgG, *n* (%)	0 (0%)	14 (12%)	0.13
**Type III cryoglobulinemia**, *n* (%)	17 (81%)	97 (80%)	1.00
Polyclonal IgG + polyclonal IgM, *n* (%)	12 (57%)	61 (50%)	0.43
Polyclonal IgG, *n* (%)	4 (19%)	18 (15%)	0.74
**Vasculitis features**			
Purpura, *n* (%)	9 (43%)	0 (0%)	<0.0001
Acrocyanosis, *n* (%)	4 (19%)	11 (9%)	0.24
Digital ischemia, *n* (%)	6 (29%)	3 (2%)	0.02
Cutaneous necrosis, *n* (%)	8 (38%)	1 (1%)	<0.0001
Ulcers, *n* (%)	8 (38%)	8 (7%)	0.0004
Livedo, *n* (%)	7 (33%)	16 (13%)	0.047
**Other manifestations**			
Arthritis and/or arthralgia, *n* (%)	18(86%)	69 (57%)	0.01
Peripheral nervous system, *n* (%)	2 (10%)	1 (1%)	0.06
Central nervous system, *n* (%)	4 (19%)	5 (4%)	0.03
Proteinuria > 1g/24h, *n* (%)	4 (19%)	18 (15%)	0.74
Proteinuria > 3 g/24h, *n* (%)	2 (10%)	14 (12%)	1.00
Hematuria, *n* (%)	3 (14%)	30 (25%)	0.41
Kidney failure, *n* (%)	1 (5%)	11 (9%)	1.00
Gastro-intestinal involvement, *n* (%)	1 (5%)	0 (0%)	0.15
**IS and immunomodulatory treatments**			
Number of IS treatments, median (range)	2 (0-7)	2 (0-6)	0.50
Corticosteroids, *n* (%)	20 (95%)	117 (97%)	0.56
Methotrexate, *n* (%)	7 (33%)	49 (40%)	0.52
Azathioprine, *n* (%)	9 (43%)	35 (29%)	0.20
Mycophenolate mofetil, *n* (%)	11 (52%)	50 (41%)	0.35
Belimumab, *n* (%)	2 (10%)	26 (21%)	0.25
Anti-TNF agents, *n* (%)	1 (5%)	2 (2%)	0.38
Cyclophosphamide, *n* (%)	7 (33%)	39 (32%)	0.92
Rituximab, *n* (%)	8 (38%)	16 (13%)	0.01
Calcineurin inhibitors, *n* (%)	2 (10%)	6 (5%)	0.34
Thalidomide, *n* (%)	1 (5%)	9 (7%)	1.00
Lenalidomide, *n* (%)	0 (0%)	1 (1%)	1.00
Plasmapheresis, *n* (%)	0 (0%)	5 (4%)	1.00

*RF* Rheumatoid factor, *SLEDAI* Systemic lupus erythematosus activity index, *CG* Cryoglobulinemia, *CryoVas* Cryoglobulinemic vasculitis, *IS* Immunosuppressive, *NA* Not applicable, *TNF* Tumor necrosis factor

### CryoVas specific treatments

Among patients with CryoVas, 13/21 (62%) patients received a specific treatment for CryoVas. Among them, 3 received corticosteroids alone, and 10 received corticosteroids with immunosuppressive drugs at first-line treatment (rituximab=4, azathioprine=3, methotrexate=2, cyclophosphamide=1, see supplemental Table 2). Cutaneous involvement motivated the initiation of these specific treatments for 11/13 (85%) patients (see supplemental Table 3).

A response to first-line treatment was observed for 12/13 (92%) patients, but relapses were observed for 3 (75%) of them. Side effects related to the specific treatment of CryoVas occurred in 3/13 patients and included cytopenia and drug-induced hepatitis (see supplemental Table 3).

## Discussion

Among the 213 SLE patients included in the present study, 66% had at least one positive test for CG, 67% of them having a persistent CG. Most of the CG were type III and were associated with low C4 levels. Among the 142 SLE CG+ patients, 15% developed a CryoVas. No death was observed in the CryoVas group and 92% of the CryoVas patients responded to the first-line treatment for CryoVas, mainly initiated based on the cutaneous indication.

The prevalence of CG in SLE patients observed in the present series is higher than that previously reported by Garcia-Carrasco et al. [16] (25%) and Karimifar et al. [10] (48%). This could be explained by the improvement in laboratory techniques for the detection of CG [21]. It is important to note that the present series is the first to study CG in SLE patients after the exclusion of HCV patients, HCV being a major cause of CG [23]. The prevalence of CG in SLE reported herein is also higher than in other connective tissue diseases. Previous series described 16% of CG in Sjogren’s syndrome [24] and 3% of CG in systemic sclerosis [25]. Moreover, 15% of the SLE CG+ and 10% of all the SLE patients from the present series developed a CryoVas, which constitutes a high rate of vasculitis for SLE patients. Previous studies reported vasculitis in 11% to 39% of SLE patients [16, 26, 27]. Interestingly, among 242 CryoVas patients, Terrier et al. [28] found only 5 (2%) patients with SLE. Overall, these results indicate that CG is frequent in SLE, with a non-negligible proportion of CryoVas. Nevertheless, CryoVas in SLE remains a scarce condition for which clinical data is poorly described in the literature.

The majority of CG were type III, even in the CryoVas group. This predominance of type III CG is consistent with previous data about CG in SLE [14, 23]. Unlike Garcia-Carrasco et al. [16], there was no significant increase in RF herein, which was positive in the cryoprecipitate or in the serum in less than 5% of SLE CG+ patients, even in patients with CryoVas. This is consistent with the results of the study by Kolopp-Sarda et al. [23], showing the absence of RF activity in 81% of mixed CG, and only 4.6% of RF activity in patients with anti-DNA antibodies. The decrease in complement fractions described herein in SLE CG+ patients is also consistent with the two main previous studies [10, 16].

Concerning SLE clinical manifestations, more pericarditis were found in the CryoVas group, a finding not reported previously, which could be due to multiple statistical testing. Similarly to Karimifar et al. [10], no association between the SLEDAI score and the presence of CG was found herein. Several studies had reported a potential association between CG and SLE nephritis [5, 29, 30], which was not observed herein, even in patients with CryoVas.

Concerning the CryoVas features, these were mainly cutaneous clinical signs. Severe manifestations such as neurological and renal involvements were rare. Surprisingly, there was more central than peripheral nervous system involvement in patients with CryoVas, which is uncommon for this vasculitis. Terrier et al. [28] found that, in patients with CryoVas, there was 52% of peripheral nervous system involvement and only 2% of central nervous system involvement. In a recent series of 71 Sjogren’s syndrome with CryoVas, patients had 2.8% and 25.4% of central and peripheral nervous system involvement, respectively [31]. Thus, it seems that the clinical phenotype of CryoVas in SLE is specific and rarely severe. Indeed, no CryoVas patient died during follow-up and only 13/21 patients received immunosuppressive therapy for CryoVas, mainly for cutaneous indication.

We observed CryoVas with low levels of cryoprecipitate. Dammacco et al. even reported an inverse correlation between cryocrit and the frequency of signs of vasculitis in patients with mixed CG [32]. Moreover, a threshold of 35 mg/L of CG has been reported for the diagnosis of CryoVas [33] with a 73% sensibility and 70% specificity (personal data). Because of the lack of correlation between the severity of CryoVas and cryoglobulin concentration between individual patients, low levels of cryoglobulins should not be ignored [34].

The management of CryoVas related to SLE remains largely unknown, as the prevalence of this condition is low. In the present series, patients received both small molecule drugs and rituximab, in addition to corticosteroids for treatment induction, with a high response rate. The 2009 EULAR guidelines recommend the use of an immunosuppressive drug for the management of non-infectious mixed CryoVas, without mentioning the particular case of CryoVas in SLE [35].

This study is, to our knowledge, the largest study addressing CG in SLE. A consecutive series of patients was included, which increased the external validity of the study. Importantly, patients with HCV, an obvious cause of CG, were excluded. There are, however, several limitations to this single-center and retrospective study. First, the inclusion of the patients was based on the laboratory results of a large university hospital (*Hospices Civils de Lyon*), possibly inducing a selection bias by capturing patients at higher risk of severe manifestations and unfavorable outcomes. Second, patients with secondary Sjogren’s syndromes were included, which can be a major cause of CG, even if the number of Sjogren’s syndromes was balanced between the two groups. Third, the distinction between features related to CG or SLE may be difficult because of the overlap in the clinical manifestations between these two conditions. Moreover, SLE may be associated with vasculitis irrespective of the presence of cryoglobulinemia. Thus, the results of the present series should be interpreted with caution in regards to the risk of information bias. Finally, all SLE patients treated in this university hospital were not tested for CG inducing a possible selection bias related to the differences in practices among physicians.

In conclusion, CG is frequent in SLE but mostly asymptomatic. CG in SLE is mainly type III and associated with more C4 decrease. CryoVas occurred in 15% of SLE CG+ patients. CryoVas in SLE appeared to be a specific condition, mainly with cutaneous manifestations and a low prevalence of neuropathy, membranoproliferative glomerulonephritis, and severe manifestations.

## Supplementary Information

**Additional file 1: Supplemental Table 1.** Cardiovascular events and severe infections in SLE patients. CMV: cytomegalovirus. **Supplemental Table 2.** Immunosuppressive and immunomodulatory treatments of SLE patients according to the presence of a cryoglobulin. IS: immunosuppressive, CG: cryoglobulinemia, TNF: tumor necrosis factor. **Supplemental Table 3.** Specific treatments of cryoglobulinemic vasculitis.

## Data Availability

The datasets used and/or analyzed during the current study are available from the corresponding author on reasonable request.

## Abbreviations

CG: Cryoglobulinemia
CryoVas: Cryoglobulinemic vasculitis
SLE: Systemic lupus erythematosus
Ig: Immunoglobulin
RF: Rheumatoid factor
ANA: Antinuclear antibodies
HIV: Human immunodeficiency virus
HBV: Hepatitis B Virus
HCV: Hepatitis C Virus
SLEDAI: Systemic lupus erythematosus activity index
SLICC: Systemic lupus international collaborating clinics
EULAR: European League Against Rheumatism
