# Supplementary material for: Cryoglobulinemia in systemic lupus erythematosus: a retrospective study of 213 patients
Source: Arthritis Res Ther. 2022 Jul 14;24:167. doi: 10.1186/s13075-022-02857-z (PMC9281087; doi:10.1186/s13075-022-02857-z)
Supplement: Supplementary file 1 — Additional file 1: Supplemental Table 1. Cardiovascular events and severe infections in SLE patients. CMV: cytomegalovirus. Supplemental Table 2. Immunosuppressive and immunomodulatory treatments of SLE patients according to the presence of a cryoglobulin. IS: immunosuppressive, CG: cryoglobulinemia, TNF: tumor necrosis factor. Supplemental Table 3. Specific treatments of cryoglobulinemic vasculitis. [file 13075_2022_2857_MOESM1_ESM.docx]

**SUPPLEMENTAL TABLES**

**Supplemental Table 1 –** Cardiovascular events and severe infections in SLE patients

|  | **SLE CG+ (n = 142)** | **SLE CG- (n = 71)** | **p-value** |
| --- | --- | --- | --- |
| **Severe infections, n (%)** | 37 (26%) | 16 (23%) | 0.56 |
| Gastrointestinal infection, n (%) | 14 (10%) | 3 (4%) | 0.15 |
| Pneumonia, n (%) | 7 (5%) | 8 (11%) | 0.09 |
| Mucocutaneous infection, n (%) | 7 (5%) | 5 (7%) | 0.54 |
| Bacteriemia, n (%) | 9 (6%) | 2 (3%) | 0.34 |
| Urinary infection, n (%) | 6 (4%) | 1 (1%) | 0.43 |
| Meningitis, n (%) | 5 (4%) | 1 (1%) | 0.67 |
| Brain abscess, n (%) | 1 (1%) | 0 (0%) |  |
| Osteoarticular infection, n (%) | 1 (1%) | 2 (3%) | 0.26 |
| Pericarditis, n (%) | 1 (1%) | 0 (0%) |  |
| CMV **^(a)^** reactivation, n (%) | 4 (3%) | 1 (1%) | 0.67 |
| Oropharyngeal infection, n (%) | 1 (1%) | 0 (0%) |  |
| Tuberculosis, n (%) | 1 (1%) | 0 (0%) |  |
| Leishmaniasis, n (%) | 0 (0%) | 1 (1%) |  |
| Retinal toxoplasmosis, n (%) | 0 (0%) | 1 (1%) |  |
| Meningococcemia, n (%) | 0 (0%) | 1 (1%) |  |
| **Cardiovascular events, n (%)** | 20 (14%) | 7 (10%) | 0.38 |
| Ischemic stroke, n (%) | 14 (10%) | 4 (6%) | 0.30 |
| Acute coronary syndrome, n (%) | 1. (4%) | 3 (4%) | 1.00 |

1. CMV: cytomegalovirus

**Supplemental Table 2** – Immunosuppressive and immunomodulatory treatments of SLE patients according to the presence of a cryoglobulin

|  | **SLE CG + (n = 142)** | **SLE CG- (n = 71)** | **p-value** |
| --- | --- | --- | --- |
| Number of IS treatments, median (range) | 2 (0-7) | 1 (0-8) | 0.05 |
| Hydroxychloroquine, n (%) | 140 (99%) | 70 (99%) | 1.00 |
| Chloroquine, n (%) | 8 (6%) | 7 (10%) | 0.36 |
| Non-steroidal anti-inflammatory, n (%) | 26 (18%) | 15 (21%) | 0.62 |
| Corticosteroids, n (%) | 137 (96%) | 60 (85%) | 0.002 |
| Methotrexate, n (%) | 56 (39%) | 26 (37%) | 0.69 |
| Azathioprine, n (%) | 44 (31%) | 25 (35%) | 0.53 |
| Mycophenolate mofetil, n (%) | 61 (43%) | 28 (39%) | 0.62 |
| Belimumab, n (%) | 28 (20%) | 7 (10%) | 0.07 |
| Anti-TNF agents, n (%) | 3 (2%) | 0 (0%) | 1.00 |
| Cyclophosphamide, n (%) | 46 (32%) | 17 (24%) | 0.20 |
| Rituximab, n (%) | 24 (17%) | 4 (6%) | 0.02 |
| Calcineurin inhibitors, n (%) | 8 (6%) | 3 (4%) | 0.76 |
| Thalidomide, n (%) | 10 (7%) | 6 (8%) | 0.71 |
| Lenalidomide, n (%) | 1 (1%) | 2 (3%) | 1.00 |
| Plasmapheresis, n (%) | 5 (4%) | 2 (3%) | 1.00 |

IS: immunosuppressive, CG: cryoglobulinemia, TNF: tumor necrosis factor

**Supplemental Table 3 –** Specific treatments of cryoglobulinemic vasculitis

|  | **Patients treated for CryoVas (n = 13)** | | |
| --- | --- | --- | --- |
|  | **First line treatment** | **Second line treatment** | **Third line treatment** |
| Rituximab, n (%) | 4 (31%) | 0 (0%) | 0 (0%) |
| Azathioprine, n (%) | 3 (23%) | 2 (50%) | 0 (0%) |
| Corticosteroids only, n (%) | 3 (23%) | 0 (0%) | 0 (0%) |
| Methotrexate, n (%) | 2 (15%) | 0 (0%) | 0 (0%) |
| Cyclophosphamide, n (%) | 1 (8%) | 0 (0%) | 1 (50%) |
| Mycophenolate – Mofetil, n (%) | 0 (0%) | 1 (25%) | 1 (50%) |
| Disulone, n (%) | 0 (0%) | 1 (25%) | 0 (0%) |
| **Indications** |  |  |  |
| Cutaneous indication, n (%) | 11 (85%) | 3 (75%) | 1 (50%) |
| Renal indication, n (%) | 1 (8%) | 0 (0%) | 1 (8%) |
| Neurological indication, n (%) | 1 (8%) | 1 (25%) | 0 (0%) |
| Gastro-intestinal indication, n (%) | 1 (8%) | 1 (8%) | 1 (8%) |
| **Response to treatment and side effects** | | | |
| Response to treatment, n (%) | 12 (92%) | 3 (75%) | 2 (100%) |
| Relapse after treatment, n (%) | 3 (23%) | 2 (50%) | 1 (50%) |
| Side effects due to treatment, n (%) | 3 (23%) | 2 (50%) | 0 (0%) |
